# Fog‐to‐Water for Water Scarcity in Climate‐Change Hazards Hotspots: Pilot Study in Southeast Asia

**DOI:** 10.1002/gch2.202000036

**Published:** 2021-03-01

**Authors:** Zaitizila Ismail, Yun Ii Go

**Affiliations:** ^1^ School of Engineering and Physical Science Heriot‐Watt University Malaysia No. 1, Jalan Venna P5/2, Precinct 5 Putrajaya 62200 Malaysia

**Keywords:** drought, economic feasibility, fog collectors, sustainability, vulnerability

## Abstract

Water is indispensable for human survival. Freshwater scarcity and unsustainable water are the main growing concerns in the world. It is estimated that about 800 million people worldwide do not have basic access to drinking water and about 2.2 billion people do not have access to safe water supply. Southeast Asia is most likely to experience water scarcity and water demand as a result of climate change. Climate change and the increasing water demand that eventually contribute to water scarcity are focused upon here. For Southeast Asia to adapt to the adverse consequences of global climate change and the growing concern of environmental water demand, fog water harvesting is considered as the most promising method to overcome water scarcity or drought. Fog water collection technique is a passive, low maintenance, and sustainable option that can supply fresh drinking water to communities where fog is a common phenomenon. Fog water harvesting system involves the use of mesh nets to collect water as fog passes through them. Only minimal cost is required for the operation and maintenance. In conclusion, fog water harvesting seems to be a promising method that can be implemented to overcome water scarcity and water demand in Southeast Asia.

## Graphical abstract

Water scarcity is the main growing concern in the world. During prolonged drought seasons, fog‐to‐water is one close‐at‐hand freshwater resource. A pilot study on fog‐to‐water solutions in climate‐change hazard hotspots in Southeast Asia is provided. The outcome makes a significant difference to sparse populations in many arid regions specially the archipelago nations in Southeast Asia.

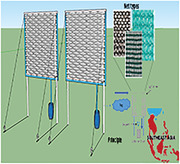

## 1 Introduction

Water is a vital resource and important for human development, health, and wellbeing. Over a billion people in the world do not have access to reliable source of clean drinking water. Climate change, increasing water scarcity, population growth, demographic changes, and urbanization seem to pose challenges for water supply systems across the world.^[^ ^1^ ^]^ Based on a report in 2017 by World Health Organization's (WHO), about at least two billion people do not have access to clean drinking water and possibly by year 2025, half of the population in the world will be living in water‐stressed areas.^[^ ^2^ ^]^ According to United Nations Sustainable Development (UNSDG), half of the population in the world has already experienced severe water scarcity for at least one month in a year, and it is expected that by 2030, about 700 million people could be influenced by intense water scarcity.^[^ ^3^ ^]^ Since the demand for water has been increasing along with growing global population and socio‐economic development especially in industrial and domestic sector, the United Nations (UN) General Assembly unanimously adopted a resolution to focus on this serious water issue for ten years (2018–2028). From 2018 to 2028, UNSDG will focus on sustainable development, management of water resources, access to water supply, and sanitation services.

Water usage has been increasing worldwide because the global water demand and the effects of climate change intensify. Water demand has been increasing and continues to increase worldwide by about 1% per year since 1980s and through rough estimation, by 2050, water demand will increase up to 20% to 30% above the current level of water usage.^[^ ^4^ ^]^ According to Ritchie and Roser, about 3.99 trillion m^3^ of freshwater withdrawals were utilized for agriculture, industry, and municipal uses in 2014, but since the amount of water usage is still ascending, the amount of clean water will further shorten in the future.^[^ ^5^ ^]^ India had the largest freshwater withdrawal of over 760 billion cubic meters per year, followed by China and United States which withdrew 600 and 480 billion m^3^, respectively in 2014.^[^ ^6^ ^]^ It has been estimated that 2.1 billion people do not have access to safe as well as clean drinking water that causes 1.2 million deaths each year which generally is three times the number of homicides and equal to the number that died in road accidents globally.^[^ ^6^ ^]^ Generally, ten billion tons of freshwater will be used up worldwide on a daily basis. United Nation estimates that 30% of the population in the world residing in 50 countries will face water scarcity or shortage. China is the country that spends the most amount of the water followed by United States of America, New Zealand, Brazil, Russia, Mexico, Canada, Australia, and England.

Water scarcity or water shortage could come in different forms and in different regions. In 2015, 2.1 billion people did not have access to basic access to safe and clean drinking water service while about 4.5 billion people did not have access to safely managed sanitation service. According to WHO/UNICEF in 2017, enormous inequalities existed between the richest and the poorest between and within countries.^[^ ^7^ ^]^ The inequality involves not only the quantity but also the quality of the supplied water. Asian countries occupy 60% of the population of the world but only possess 36% of the available water. Meanwhile North, South, and Central America only have 14% of the population of the world but they have 41% of accessible water.^[^ ^8^, ^9^ ^]^ In 2017, 673 million people around the world (around 9% of the global population) still defecated in open, with the majority in Southern Asia. The wealthier or non‐slum households usually only need to pay a little for high level services while the poor need to pay a higher price for a service of similar or lower quality, which includes having access to water and sanitation facilities. The water scarcity is experienced in many regions since water naturally is not evenly distributed in space and time.^[^ ^10^ ^]^ Report by WSSC in 1999 stated that urban residents in the United States typically only need to pay $ 0.40–0.80 m^−3^ for excellent high‐quality water while residents in Jakarta (Indonesia) and Lima (Peru) are forced to purchase water for a price 20–50 times higher than residents that are connected to the city system.^[^ ^11^ ^]^

**Table** **1** shows the water withdrawal by the continent as reported by previous research.^[^ ^12^, ^13^, ^14^, ^15^, ^16^, ^17^, ^18^, ^19^ ^]^ Asia shows the highest water withdrawal over the other continents at 23 813 km^3^ per year followed by South America, 20 760 km^3^ per year. The smallest volumes of water withdrawal are by Europe and Australia with Oceania at respectively 1200 and 200 km^3^ per year. The enormous difference in water withdrawal is due to various growing seasons of different crops that vary spatially depending on cropping practices and climatic conditions.^[^ ^20^ ^]^ Typically, a massive water withdrawal is required for agriculture sectors usually in paddy (rice) as well as non‐paddy crop types which consume nearly 70% of the total water withdrawal before followed by industrial and domestic water at about 18% and 13% of the total water withdrawal.^[^ ^21^, ^22^ ^]^ In addition, in terms of crop intensities, the water withdrawal is also influenced by the average number of crops growing within a year since some crops can only be harvested once a year while some can be harvested twice a year.

**Table 1:** Water withdrawal by region^[^ ^12^, ^13^, ^14^, ^15^, ^16^, ^17^, ^18^, ^19^ ^]^

	Continental Runoff [km³ per year]							
	Study							
Region	Vörösmarty et al.^[^ ^12^ ^]^	Cosgrove and Rijsberman^[^ ^13^ ^]^	Hanasaki et al.^[^ ^14^ ^]^	Döll and Siebert^[^ ^15^ ^]^	FAO^[^ ^16^ ^]^	Shiklomanov^[^ ^17^ ^]^	Oki et al.^[^ ^18^ ^]^	Wada et al.^[^ ^19^ ^]^
Africa	4520	6470	1400	1400	1800	4050	5815	2132
Asia	13 700	23 813	21 400	18 800	19 400	13 510	13 014	22 948
Europe	2770	3588	1600	1200	1300	2900	6286	3922
Oceania/Australia	714	2680	200	300	200	2400	1912	2630
North America	5890	5145	2400	1900	2000	7890	5345	6225
South America	11 700	20 760	1200	1000	1900	12 030	14 906	1646

Climate change is impacting the global community diversely at different severity and scale from food security, water supply, extreme weather, crop growing and harvesting patterns, altered natural habitat and coastline, health, etc. Intergovernmental panel on climate change (IPCC) reported that greenhouse gasses that accelerate climate change need to be reduced to net zero emissions by 2025 globally. Southeast Asia (SEA) has been identified as one of the regions which are greatly impacted by climate change, especially vulnerable communities at the hazard hotspots. Thus, this paper is aimed to provide a comprehensive review on the impact of climate change on water scarcity issue in Southeast Asia and the feasibility of fog‐to‐water as an alternative solution for water supply. This paper is a pilot study to carry out an exploratory, techno‐socio‐economic study on the feasibility of fog‐to‐water solution including resource assessment, water withdrawal, water accessibility, water stress, climate risk assessment, dominate hazards, fog water harvesting method, collection rate, economics of fog collection, societal impact etc. This paper contributes to water adaption planning, climate change mitigation scheme, and policy development for government to strategize the assistive scheme and adaption measures in Southeast Asia.

## 2 SEA Countries

Asia is the most populated continent among all the seven continents in the world including Africa, Australia, Antarctica, Europe, North America, and South America whereby the population of Asia makes up 60% of the total human population. Southeast Asia is located in the Asian continent and currently comprises 11 countries, namely Brunei, Cambodia, East Timor (Timor‐Leste), Philippines, Indonesia, Laos, Malaysia, Myanmar (Burma), Singapore, Thailand, and Vietnam. The population of Southeast Asian countries continues to increase from 1950 until now whereby the population rose from 480 million in 1995 to 593 million in 2010. The population in Asia makes up 8.58% of the total world population in which the population has doubled in 32 years since 1963 and rose drastically to 67% from initial population of 287 million in 1970 over the period of a quarter of the century.^[^ ^23^, ^24^ ^]^

Urbanization in South East Asia has been increasing rapidly at an impressive pace over the last few decades. Developments in Southeast Asia have been accompanied by extraordinary growth in the population of Southeast Asian countries especially during the second half of the 20th century. Human population for countries in Southeast Asia has grown from 80 to 530 million whereby the extraordinary growth might be due to remarkable as well as rapid declination in mortality of the citizens. The tremendous changes in population size and growth are closely intertwined with the economic, social, as well as political transformations of the country.^[^ ^25^ ^]^ Indonesia has the highest population followed by Philippines and Vietnam. Undoubtedly, the high population in these three regions has induced some concerns and eventually influence the population policy especially policy related to migration.^[^ ^26^ ^]^ Interestingly, population growth of 29% or 140 million in Southeast Asian countries over the two decades grants the countries an intermediate position among the ranking of countries with highest population and at the same time it has become almost twice as rapid as China. **Table** **2** lists the land area for every country in Southeast Asia along with the estimation of population by UN.^[^ ^27^ ^]^ The population in every county is rapidly increasing in every decade. Rapid increment in the human population in the recent decades takes place mostly in agricultural regions such as East and Central Java, the Red River delta of northern Vietnam, as well as Visayan region in Philippines in which all the regions are considered as frontier rice growing regions.

**Table 2:** Land area and human population based on UN estimation^[^ ^27^ ^]^

Countries	Land area [km^2^]	UN population estimates [thousands]			
	2015	1980	1990	2000	2010
Brunei	5769	189	252	327	399
Cambodia	181 035	6506	9532	12 447	14 138
Indonesia	1 913 579	150 820	184 346	213 395	239 871
Laos	236 800	3235	4192	5317	6201
Malaysia	330 290	13 833	18 209	23 415	28 401
Myanmar	676 577	32 865	39 268	44 958	47 963
Philippines	300 000	47 064	61 629	77 310	93 261
Singapore	719	2415	3017	3919	5086
Thailand	513 120	47 483	57 072	63 155	69 122
Timor‐Leste	14 870	581	743	830	1124
Vietnam	330 951	54 023	67 102	78 758	87 848
Southeast Asia	4 503 710	359 014	445 362	523 831	593 414

## 3 Water in Southeast Asia Development

Water is a necessity for life and plays a crucial role in sustaining high quality life and for social as well as economic development. Over the recent years, water usage in Southeast Asia has been increasing due to its rising demand for agricultural, industrial, municipal, and other uses in which agricultural purpose alone has consumed more than two‐thirds of the overall water supply. In addition, the changes in living standards as well as lifestyle and drastic rise in population might also be the reason behind the steady increment in the total water consumption in the Southeast Asia. Report by FAO in 2015 shows a 31% declination for estimated total renewable water resources per capita from an average of 23 640 m^3^ per inhabitant per year in 1992 to 16 272.63 m^3^ per inhabitant per year in 2014 across Southeast Asia.^[^ ^28^ ^]^ Southeast Asia lies in region with humid tropical climate that can be identified by the presence of seasonal monsoon rains, floods, and river overflow. Many cities in Southeast Asia are located on or beside major rivers such as Vientiane and Phnom Penh on the Mekong River, Hanoi on the Red River as well as Bangkok on the Chao Phraya River. Water sources have always been contemplated as central hotspot for daily living and economic activities of human being whereby nowadays water sources such as river are often materially transformed to suit the development of human being. River modification program or project in a large scale have resulted in channelized waterways, the flows of the river being regulated through dams, large scale of irrigation as well as extraction project, flood prevention works and inter‐basin diversion project.^[^ ^29^ ^]^

In addition, urban water problems in Southeast Asia come under two contradictory categories which are problems caused by economic development activities and problems caused by underdevelopment of available resources.^[^ ^30^ ^]^ Special care and consideration need to be taken for the two particular problems so that the water problems in Southeast Asia can be restrained while sustaining a water resources development in the region. The underdevelopment of water resources might be related to two important aspects which are the climatic variability and the size of the river basins. Climatic variability is known to occur frequently in Southeast Asia whereby the varieties of the climate can be divided into three basic kinds including tropical, dry, and temperate climate. Usually, the temperature for all regions in Southeast Asia is above 25 °C throughout the year while being strongly influenced by Asian monsoons.^[^ ^31^ **^]^** Asian monsoons bring significant amount of rainfall to parts of Southeast Asia. Although climatic variability contributes to underdevelopment of water resource, the size of river basins also affects the urban water problems in Southeast Asia. There are several important river systems in Southeast Asia especially in Indonesia and Thailand. However, most urban cities or centers are located near or in very small river basins that often lead to several serious problems. Urbanization in small river basins is associated with the occurrence of river pollution and flooding. The urban activities often impose with overextraction of groundwater and subsequently induce ground subsidence.^[^ ^32^ ^]^

**Figure 1:**
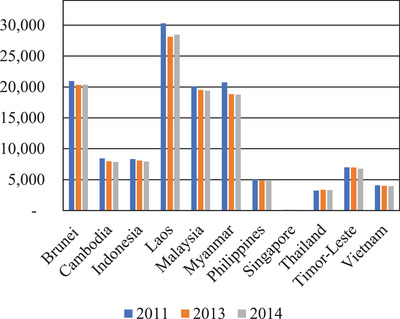
Internal renewable freshwater resources per capita cubic (m^3^ per inhabitant per year) in Southeast Asia (2011, 2013, 2014).^[^ ^33^, ^34^, ^35^ ^]^

Water accessibility or water availability in Southeast Asia has declined year by year. **Table** **3** shows the data on internal freshwater resources that derived from the estimation of runoff into rivers and recharge of groundwater in 2013, 2015, and 2016.^[^ ^33^, ^34^, ^35^ ^]^ Through **Figure** **1**, declination in the internal renewable freshwater resources per capita for the countries of Southeast Asia for three years (2011, 2013, and 2014) can be clearly observed.^[^ ^33^, ^34^, ^35^ ^]^ Notably, Singapore is the country with the lowest internal renewable freshwater resources per capita cubic meters that make Singapore as the only country which has been categorized as country with water stress. Water stress is defined by estimating the number of people per flow unit annually or based on the ratio of freshwater withdrawals to renewable freshwater resources.^[^ ^5^ ^]^

**Table 3:** Water in Southeast Asia (World bank 2013, 2015, 2016)^[^ ^33^, ^34^, ^35^ ^]^

Region	Internal renewable freshwater resources			Access to improved water source			Access to improves sanitation facilities			Urban population		
	per capita cubic meters (m^3^ per inhabitant per year)			% of total population			% of total population			% growth		
	2011	2013	2014	2010	2012	2015	2010	2012	2015	1990–2011	2012–2013	2013–2014
Brunei	20 939	20 345	20 364	–	–	–	–	–	–	2.2	1.8	1.8
Cambodia	8431	7968	7868	64	71	76	31	37	42	2.1	2.7	2.6
Indonesia	8332	8080	7935	82	85	87	54	59	61	2.5	2.7	2.7
Laos	30 280	28 125	28 463	67	72	76	63	65	71	4.7	4.9	4.6
Malaysia	20 098	19 517	19 397	100	100	98	96	96	96	2.5	2.7	2.5
Myanmar	20 750	18 832	18 770	83	86	81	76	77	80	2.5	2.5	2.5
Philippines	5050	4868	4832	92	92	92	74	74	74	2.2	1.3	1.3
Singapore	116	111	110	100	100	100	100	100	100	2.1	1.6	1.3
Thailand	3229	3350	3315	96	96	98	96	93	93	1.7	3	2.9
Timor‐Leste	6986	6961	6777	69	71	72	47	39	41	4.2	4.8	4.7
Vietnam	4092	4006	3961	95	95	98	76	75	78	3.1	3.1	3.0

There are four stress levels that are originally determined through per capita water availability based on a comparison of national resources availability data with assessment of whether a country was experiencing water related problems whereby the results are as shown in **Table** **4**.^[^ ^36^ ^]^ From the determined stress level, Singapore can be categorized as country with extreme stress in per capita water availability [m^3^/c/y] in which occurrence of the water stress is not because of lack of rainfall (2400 mm per year), but because of the restricted land area to be used as rainfall storage area.^[^ ^37^ ^]^ Other countries that face economic water scarcity include Thailand, Vietnam, and Timor‐Leste. Although Singapore face water stress with population density of 7400 persons per km^2^, but Singapore has the highest figure for percentage of population with access to improved water source and sanitation facilities followed by Malaysia.^[^ ^38^ ^]^ The high percentage of population with access to improved water source is due to a number of recent reforms done by local environment governance including increased decentralization, community, and private sector participation as well as the privatization of water facilities.^[^ ^39^ ^]^ Over the last decade, all the countries in Southeast Asia have shown a slight increase in the percentage of population with access to improved water source and sanitation facilities.^[^ ^40^, ^41^ ^]^

**Table 4:** Water resources index classes^[^ ^36^ ^]^

Per capita water availability (m^3^/c/y)	Stress level
>1700	No stress
1000–1700	Moderate stress
500–1000	High stress
<500	Extreme stress

**Figure 2:**
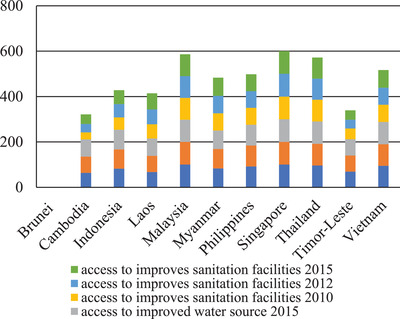
Percentage in total population in access water source and sanitation facilities in Southeast Asia (2011, 2013, 2014).^[^ ^33^, ^34^, ^35^ ^]^

While the entire population in Singapore and Malaysia have access to water sanitation facilities, in countries such as Cambodia and Timor‐Leste however have the lowest figures for percentage of population with access to improved water source and sanitation facilities as seen in **Figure** **2** whereby only less than half population in both countries have access to improved water source and sanitation facilities.^[^ ^33^, ^34^, ^35^ ^]^ Almost more than 39% of total population in Indonesia do not have access to water sanitation facilities, 29% do not have safe access to sanitation facilities in the Laos while 26% and 22% do not have access to sanitation facilities in Philippines and Vietnam respectively. Thailand has shown an improvement with reaching almost 100% in population having access to water source and sanitation facilities. An improvement in percentages figures with a jump from 76% in 2010 to 80% in 2015 also recorded for Myanmar.

**Figure 3:**
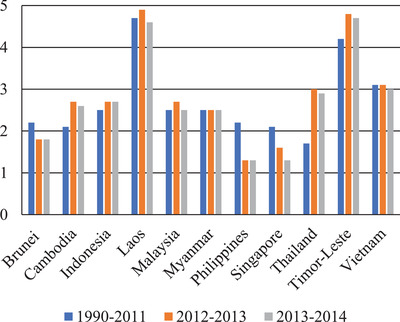
Percentage in total population in urban growth in Southeast Asia (2011, 2013, 2014).^[^ ^33^, ^34^, ^35^ ^]^

Growth and development usually have been centered in the major metropolitan centers.^[^ ^42^ ^]^ Laos and Timor‐Leste possess the highest percentage of urban population growth population from 1990 to 2014 as illustrated in **Figure** **3**.^[^ ^33^, ^34^, ^35^ ^]^ Even though both of the countries have increasing percentage in urban population growth, but Laos and Timor‐Leste are also the countries with the highest percent of their total population living in poverty for about 39%.^[^ ^43^ ^]^ According to Mills and Pernia, the continuation in urbanization and urban population growth will usually tend to lead to urban phenomenon which resulted to increment in poverty.^[^ ^44^ ^]^

Water resources are usually stressed and strained as a result of population growth, rapid urbanization, expansion of irrigated agriculture and industrialization. The situation is worsened due to global emission of greenhouse gasses and climate variability that bring about water‐related disaster such as droughts, flooding and the rise in sea level. Poor governance also can contribute to freshwater stress. Government need to collaborate with private sector in water utilities issues to avoid serious water stress problems. Many policies and regulatory bodies are aware of the growing problems of freshwater stress and water scarcity. If the water scarcity issue is not restrained, many rivers and lakes will eventually end up being polluted from animal as well as human wastes, metal from mining, and various chemical wastes.

## 4 Climate Change and Water Assessment

Aside from increasing population and economic development, countries in Southeast Asia are currently facing the consequences of climate change on the overall availability of water. The climate variations affect the availability water while continuously rises the water demand in the past 50 years across the world.^[^ ^45^, ^46^, ^47^ ^]^ Even though Southeast Asia receives abundant rainfall and has been blessed with abundant water resources, unfortunately climate change including rises in temperature, increasing sea level along with impacts on the coastal area, thermal alterations, pollution, species extinctions and changes in precipitation takes its toll on freshwater.^[^ ^48^ ^]^ The negative impact due to climate change directly affects several physical, biological and ecosystem of the respective area whereby the possible negative impacts including poleward or even altitudinal shifts in the livable range for plant and animal, declination of some plant and animal populations, earlier flowering of trees, earlier emergence of insects as well as earlier egg‐laying in birds.^[^ ^49^, ^50^ ^]^ The possible occurrence of negative impacts due to climate change is supported by Nijssen et al. who identify one of climate change effect, the changes in the greenhouse gas concentration whereby the incorporation of modern land surface parameterizations is required.^[^ ^51^ ^]^ The climate change is expected to cause a 1.1 to 6.4 °C increment in average temperature along with possible shrinkage of glaciers, earlier break‐up of ice on rivers and lakes and thawing of permafrost.^[^ ^52^ ^]^

Southeast Asia is exposed to a range of climate hazards and vulnerabilities including frequent heatwaves, heavy rainfall events, cyclone, floods, droughts, storms, land degradation, and biodiversity loss. The climate hazards can eventually affect relations between states through humanitarian crises, migration, greater dependency on imports of vitals goods, and even creating conflict.^[^ ^53^, ^54^, ^55^ ^]^ The climate hazard will certainly have negative impact on agricultural yields, biodiversity, forest harvests, and availability of clean water. The majority of countries in Southeast Asia have a wet and dry seasons caused by seasonal shift in winds as well as monsoon with exception for only Northern Vietnam and Myanmar Himalaya whereby both regions have a subtropical climate which has cold winter with snow. According to the Global Climate Risk Index, four of the world's ten most climate change affected countries from 1996 to 2015 are located in Southeast Asia which include Myanmar, Philippines, Thailand, and Vietnam. Nonetheless, only Philippines in Southeast Asia are being seriously affected by climate change in 2018.^[^ ^56^, ^57^ ^]^

According to International Panel on Climate Change (IPCC), Southeast Asia is vulnerable to a rise in the sea‐level up to 70 cm by 2100 from the sea level back in year 1990 while in Indonesia, Philippines, Thailand, and Vietnam, the annual temperatures could possibly rise by 4.8 °C.^[^ ^58^, ^59^ ^]^ The increasing sea level could force up to 21 million people in Southeast Asia including 10% of residents in Mekong Delta be displaced to new location.^[^ ^60^ ^]^ The rise of sea‐level from the melting of ice will lead to the intrusion of saltwater into coastal and groundwater resources eventually threaten the supplies of freshwater for drinking and irrigation purpose. Southeast Asia has a special geography that makes the region particularly sensitive and prone to be severely affected by climate change that may induce many risks on climate related developments.

**Figure 4:**
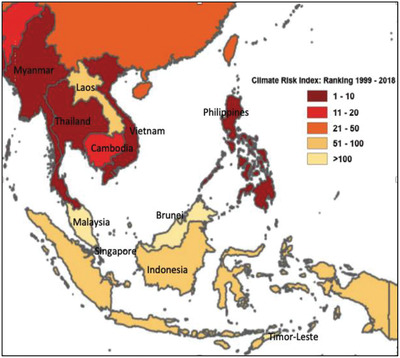
Climate risk index in Southeast Asia: Ranking for 1999–2018 (Eckstein et al. 2019).^[^ ^57^ ^]^

**Figure** **4** and **Table** **5** show the overall countries that tend to be affected the most by extreme weather events, climate risk index (CRI), gross domestic product (GDP) as well as purchasing power parity (PPP) in Southeast Asia. Myanmar, Philippines, Vietnam, and Thailand are the countries that seem to be most affected by climate change in Southeast Asia in 2018. In 2008, Myanmar experienced Cyclone Nargis which was responsible for estimated loss of 140 000 lives with ≈2.4 million people losing their property.^[^ ^62^, ^63^ ^]^ Extreme flood in current wet season has forced over 190 000 people to seek emergency shelter while creating damages to homes, schools, and farms.

**Table 5:** Ranking climate risk (RCI), dominant hazards of Southeast countries, climate risk index for 2018; Kreft et al., 2016;^[^ ^60^ ^]^ Eckstein et al. 2019;^[^ ^57^ ^]^ Thinkhazard 2017^[^ ^61^ ^]^

RCI	Country	Dominant hazards	CRI score	Fatalities in 2018 (Rank)	Fatalities per 100 000 inhabitants (Rank)	Losses in million US$ (PPP) (Rank)	Losses per unit GDP in % (Rank)
1	Myanmar	Floods, landslides, cyclone	53.83	20	43	59	79
2	Philippines	Cyclones, landsides, floods, droughts, earthquake, tsunami, volcano	11.17	4	14	7	14
3	Vietnam	Droughts, typhoon, cyclones, sea level rise, floods, landslides	26.17	9	33	18	32
4	Thailand	Sea level rise, floods, drought, cyclones	68.83	25	59	60	105
5	Cambodia	Floods, cyclones	47.67	29	17	79	72
6	Indonesia	Droughts, floods, landslides, sea level rise, earthquake, tsunami, volcano, cyclone	68.17	11	74	42	104
7	Laos	Floods, landslides, cyclone	35.5	26	8	69	51
8	Malaysia	Droughts, floods	84.83	45	61	96	123
9	Brunei	Floods	125	115	115	135	135
10	Singapore	Floods	125	115	115	135	135

Philippines that consists of over 7000 islands is particularly vulnerable to storms and is the second most risk‐prone country in the Southeast Asia.^[^ ^64^ ^]^ Typhoon Haiyan, the strongest storm ever recorded to strike the land, hit hard on one of the Philippines provinces, Leyte in November 2013. People in Leyte endured the estimated 1 min sustained wind gusts of 315 km h^−1^ which killed at least 10 000 people and brought about extensive economic damage to Leyte.^[^ ^65^ ^]^ Philippines could possibly suffer an overall productivity loss, lowering GDP from 17% to 58% along with increasing direct damage to housing if adaptive measures are not taken.^[^ ^66^ ^]^

Vietnam is a densely populated coastal country with diversity that increases the chances of the country to be hit by cyclone, the rise in the sea level, drought, floods, typhoon, and landslide which eventually make it as one of the most hazard‐prone country in the world. According to United States Agency International Development (USAID), climate change had claimed 9500 lives that were responsible for losses equal to 1.5% of annual GDP between 2001 and 2010.^[^ ^67^ ^]^ As seen in Table 5, in 2018, GDP increased to 32% and is expected to worsen in coming years. The annual mean temperatures seem to rise since 1960 with increment of +0.5 °C whereby the rate of increment becomes more rapid in the dry season and will possibly increases by 1–2 °C in 2050. Mekong River Delta has always been the largest agriculture and aquaculture production ground in the Vietnam. Recently, climate changes induced water shortages and altered river flows as well as groundwater level. In 2016, the worst drought in 90 years with the lowest water level in Mekong River recorded the eventually reduced water supplies for irrigation, downstream river basins, and hydropower reservoirs purposes.^[^ ^68^, ^69^, ^70^ ^]^

Thailand is the home to 66.7 million people and the largest exporter of rice in the world. The occurrence of climate change mainly threatens three major sectors that affect the overall economy of Thailand which are the agriculture, tourism, and trading. In the last decade, weather patterns in Thailand have fluctuated from severe droughts to severe floods. In 2005 and 2008, over ten million of population in Thailand suffered from drought and water shortages since the rainfall was below a normal level.^[^ ^71^ ^]^ National Hydro informatics and Climate Data Center (NHC) reported that water in reservoirs nationwide was at critically low levels due to prolonged droughts between 2015 and 2016. Meanwhile in 2011, Thailand experienced the worst flood with a cost of USD 46 billion employed to repair and bring about rehabilitation nationally. The flood affected more than 13 million people and resulted in over 680 deaths.^[^ ^72^ ^]^

Indonesia is the fourth most populated country in the world and the world's largest island country. Indonesia has two seasons which are wet and dry season. World Bank analysis report revealed that Indonesia faces high mortality risk due to multiple hazards including droughts, floods, earthquakes, landslides, and tsunami. The climate variability is changing the frequency as well as the intensity of weather that brings natural disasters to Indonesia while threatening the coastal population and infrastructure of Indonesia. According to Oktaviani et al., Indonesia is predicted to experience a rise in overall temperature for 0.8 °C by 2030.^[^ ^73^ ^]^ The increment in temperature will affect Indonesia through a rise in the sea level. Indonesia is one of the countries in the world that is most vulnerable to a rise in sea level as 42 million people living on low‐lying land less than 10 m above sea level and about 5.9 million people will be affected every year by 2100.^[^ ^74^, ^75^ ^]^ Moreover, climate change also causes droughts especially during El Nino that occurred once every 4 years before 1960 but now is being reported to occur every 3 years.^[^ ^76^ ^]^

Climate change increases water resources stress in Southeast Asia. Although climate change increases runoff, the runoff is not very beneficial practically because the increasing runoff tend to come during the wet season instead the dry season which makes the extra water become unavailable during the dry season. Climate change brings tremendous effect to water resources in Southeast Asia which includes droughts, floods, rise in sea level, increasing water resources stress and affecting the population in terms of quality of life, safety, as well as health of the people. After the occurrence of natural disaster due to climate change, the affected region will face shortage in water supply and lack of clean water. The lack of water resources directly affects the economy, environment, and society of a population. Agricultural producers, food security, and forestry are particularly in danger and suffer greatly from the climate change especially in deficiency of water resources. Recently in June 2019, an uncontrolled fire also known as Black Summer took placed in Australia. The fires rapidly spread across all states whereby an area about the size of South Korea of roughly 25.5 million acres had been burned.^[^ ^77^ ^]^ The fire destroyed buildings, killed people and in rough estimation, about one billion animals have been killed while some endangered species may had been be driven into extinction.^[^ ^78^, ^79^ ^]^ The hot and dry seasons that spread across the country made forests become drier and more capable of inducing bushfires more frequently with greater intensity.

Climate change, population growth, and urban development increases competition for obtaining fresh water that eventually causes water shortages. To alleviate the water stress, various solutions and methods have revealed approaches to cope with water scarcity and had been successfully applied worldwide. Ocean desalination, rainwater collection, and fog harvesting are some of the methods for water harvesting. Seawater desalination technologies separates saline seawater into two stream which are freshwater stream that contains a low concentration of dissolved salt and a concentrated brine stream.^[^ ^80^ ^]^ Turning seawater into freshwater is the most favored water harvesting technique since the Earth is covered in 97% sea water. However, many countries unable to afford the ocean desalination technology as a freshwater resource due to the high desalination costs.^[^ ^81^ ^]^ The cost to build a 5760 m^3^ per day multiple‐effect distillation thermos‐compression desalination plant for $ 8.3 M or $ 1.54 M per 1000 m^3^.^[^ ^82^ ^]^ Desalination pollutes the air from emission of carbon dioxide (CO_2_), carbon monoxide (CO) and nitrogen oxides (NO) due to burning of natural gas fuel to produce desalted water, contributing to the greenhouse gases that cause a climate change. In addition, the concentrated salt stream and chemical used in ocean desalination method might be a huge environmental risk to the ocean and marine environment if the waste products from the technique are not properly managed.^[^ ^83^ ^]^ The thermally driven multi‐stage flash desalting process has negative effects on marine environment by i) affecting the seawater intake and impingement of marine species on intake screens, ii) brine discharge of high salinity brine with chemicals used in pretreating the feedwater, and iii) thermal discharge to the sea by high‐temperature brine and cooling seawater and energy‐intensive with costly process.^[^ ^84^ ^]^

Capacitive deionization (CDI) is an upcoming brackish water desalination technology. This technology uses the science of electrodes where the ions are removed from the water by electrostatically absorbing them onto porous electrodes. Another desalination process is multi‐effect distillation (MED), the science of evaporation and condensation is used in this process.^[^ ^85^ ^]^ The average cost of MED is about $1.12 m^−3^.^[^ ^86^ ^]^ The major advantage is of this process is that it uses lesser energy, has better overall heat‐transfer coefficients, and low operating steam temperature compared to the Multistage flash desalination (MSF). The cost of MSF is about $0.89 m^−3^ in Qatar.^[^ ^86^ ^]^

Another means of desalination is membrane technologies. There are two types of membrane processes: 1) water is normally transported across the membrane as it is driven by pressure and 2) the solute is transported across the membrane as it is driven by electrical potential. Reverse osmosis is the process of separating saline water via a semipermeable membrane with the help of a pressure profile. The pressure applied needs to exceed the osmotic pressure to allow the process of osmosis to happen. The most crucial component of the reverse osmosis is the membrane; the semipermeable membrane has a characteristic where it allows only water to pass through leaving the solute behind. The characteristics that are needed for a reverse osmosis membrane includes the strength to endure the operating pressure, a chemical surface that would attract water, and pores to transport the water.^[^ ^85^ ^]^ The cost of reverse osmosis can range from $0.5 to $2.5 m^−3^.^[^ ^86^ ^]^ Electrodialysis (ED) is a membrane desalination process that eliminates salt water from feed water with the help of electrodes. ED is a desalination process that has been used for many years which utilizes electrochemical process for the separation of the ions across a charged membrane.^[^ ^85^ ^]^

The rainwater harvesting method on the other hand is a technique of capturing storm water runoff which is usually from the roofs of houses, land surface, road surface or rock catchments, and the storage tank for later use.^[^ ^87^ ^]^ 1 mm of harvested rainwater is equivalent to 1 L of water per square meter. Harvested rainwater will usually be used for rainfed agriculture or water supply for households.^[^ ^88^ ^]^ Even though rainwater harvesting is a simple, renewable source and inexpensive, this technique is unpractical for regions that have low rainfall since the concept for rainwater harvesting includes the catchment of rainwater. Rainwater may be polluted by bacteria, metals, suspended solids, and total Kjeldahl nitrogen that require a disinfect procedure to be done before the harvested water can be utilized.^[^ ^89^ ^]^ Although it is imperative to analyze the cost of producing water from fog, it is important to consider that it cannot be compared directly with other sources of water, such as desalination, because of the differences in scale of the two technologies.

Dew harvesting is another form of water harvesting. There are three ways for dew harvesting which are 1) passive (radiative) cooling condenser, 2) solar‐regenerated desiccant, and 3) water harvesting from air using active cooling condensation technology. The principle of radiative cooling condenser is inspired by the formation of dew on leaves. The formation of dew is caused by radiation that occurs on the surface of the materials. A dew formation is highly dependent on the ability of the surface to cool without external energy. The most crucial element is the power gradient between the condenser outgoing radiative power and the sky radiative power.^[^ ^90^ ^]^

Solar regenerated desiccant in water harvesting is another form of dew harvesting. Materials like silica gel, zeolites, and CaCl_2_ are hygroscopic and are suitable for desiccant materials because they are moisture absorbent. Desiccant beds are widely used to harvest atmospheric water these days. The first stage of this process is the absorption of water by the desiccant bed from humid air at night. The next stage is the heating of the desiccant bed with solar radiation to allow desorption during the day, at this stage the desiccant regenerates and drives the water vapor out. The final stage is when the evaporated water is condensed into droplets that are collected in a tank. There are several collector designs that can be improve the desiccant performance namely the glass pyramid collector, trapezoidal prism, Corrugated surface and solar glass desiccant box type system.^[^ ^90^ ^]^

An active harvester is using active cooling condensation technology to harvest water from air. There are several active harvester technologies that are prominent these days. The first one is dehumidifier using selective membrane. To minimize the power consumption of the process of dehumidification, selective membranes have been used to separate the water vapor component before the cooling and condensation process. This will eliminate the need to cool the other atmospheric gases. The main element of the system is the membrane driven by concentration gradient imposed by the vacuum pump that only allows water to pass through.^[^ ^90^ ^]^

Atmospheric water harvesting integrated with air conditioning system and condensing coil is another active harvester. This condensing system uses a conventional reverse Rankine cycle to operate. It operates in the same way as a dehumidifier where a passage of moist air passes over a coil which is cooled by a refrigerant which causes the water vapor to condense. Thermoelectric cooling is an option for water harvesting which as well is looked into as an alternative for the conventional Rankine cycle for water harvesting.^[^ ^90^ ^]^

Fog water harvesting is an economical, passive, and sustainable option with minimal maintenance to supply drinking water, supplement rainfall in arid climates for reforestation as well as water usage for the communities.^[^ ^91^ ^]^ Fog is defined as a mass of water droplets suspended in the atmosphere in the vicinity of the surface of the Earth that will usually affect visibility or generally, a cloud that touches the ground.^[^ ^92^, ^93^ ^]^ Fog water harvesting or collection is a suitable method to be used in areas with high humidity ratio. Fog water harvesting technique has been studied for over 20 countries across the six continents depending on the regional conditions whereby research suggested that fog water harvesting or collection is the best idea or solution to overcome water supply issues since the source, fog can be collected even in arid region. More in‐depth details in fog water harvesting will be discussed in the next section.

## 5 Fog Water Harvesting and Technical Feasibility

Fog water harvesting has been conducted in South Africa from 1901 to 1904 in order to investigate the amount of water that could be collected from fog as a natural supplement for another alternative for water resources.^[^ ^94^ ^]^ Technologies on fog water harvesting systems have been improved while experiencing major development after fog water harvesting method was introduced in the mid‐20th century.^[^ ^95^ ^]^ Some studies on fog water harvesting included two rain gauges to determine the volume of fog deposition. Later research concentrated on the size as well as the composition of material utilized to make the fog collection nets, direction along with the angle of installation for the fog collector, intensity of the wind, height above ground, mesh material and climate as well as the topography of the area involved. There is a timeline in the history mentioning several fog and dew water collection methods that were practiced in arid as well as semi‐arid areas. Fog can be found all over the world even in the desert particularly in the early morning and late evening hours. There are many research works that have been conducted on fog water harvesting in arid and semi‐arid regions.^[^ ^96^, ^97^, ^98^, ^99^ ^]^ Previous research reported that fog water collection or dew collection are effective even in dryland areas with less annual rain.

Fog is a type of alternative water source. Fog is very low‐lying cloud and made up of millions of tiny water droplets or ice crystals. Officially fog is defined as a mass of water droplets suspended in the atmosphere in the vicinity of the earth's surface that effect the visibility or a cloud that is touching the ground.^[^ ^93^, ^100^ ^]^ Fog droplets have diameters from about 1 to 30 µm and even 0.5 mm with fall velocities of less than 1 to ≈5 cm s^−1^ that originated from water lost through evapotranspiration which cause masses of humid near over land as well as air.^[^ ^101^, ^102^ ^]^ Air in fog has a relative humidity above 95% while the density of water in fog is 0.05–0.5 gm^−3^. Usually, the formation of fog is the highest among mountainous areas near the coast. Fog water harvesting is considered as a vital water source in desert environments. Generally, fog droplets are collected through a simple surface impaction process. The fog droplets are intercepted as the air passes through the prepared mesh that usually suspended above the ground between two vertical posts whereby the mesh is positioned at a right angle to the fog bearing wind. Then fog droplets coalesce and form larger water droplets on the mesh fabric, the harvested water will run down into a collecting drain or tank system.^[^ ^103^ ^]^

The fog collectors consist of a flat rectangular net which is supported by a post at both ends of the net. The net is arranged perpendicularly to the direction of the predominant wind. The number of modules is normally chosen based on the topography and geographical requirements of the locations. The modules of the netting can be simple, which is a panel with a surface area of 48 m^2^ or more complex structure which is made up of a series of collection panels arranged together. Droplets are captured as the fog droplets are intercepted with the mesh that is stretched out fronting the dominant direction of wind flow. The fog droplets amalgamate and produce bigger water droplets etched to the mesh. Once the panels are installed based on the location's demands the troughs below the panel are connected to a network of pipes that will transport the water to the storage tank. From the storage tank the water can be distributed by several methods based on how the water is going to be used.^[^ ^104^ ^]^

Studies on fog water harvesting have been carried out over the last few decades and considered as a viable unconventional water source in many parts of the world. Water harvested from fog water harvesting capable of alleviating shortages of water for domestic, small‐scale agricultural and reforestation purposes. Numerous fog water harvesting research have been done in many regions whereby extensive discussions are reported along with achieved results, sustainability factors, pro and cons of the technology of the fog water harvesting. **Table** **6** presents fog harvesting's technical feasibility, water yield, and parameters investigated in the research.

**Table 6:** Fog harvesting's technical feasibility, water yield, and parameters investigated in the research

No	Title		Technical Feasibility		Yield		Parameters	Ref
1	Electrostatically driven fog collection using space charge injection.		Adopt electrical forces to overcome aerodynamic drag forces. Use ion emitter to introduce space charge into the fog and direct them to collector via imposed electric field.		30 min of exposure to corona discharge resulted in 30 mL of water collection.		– Collection efficiency, area. – Acceleration phase, electric field. – Terminal velocities, corona discharge.	^[^ ^105^ ^]^
2	Experimental study of fog water harvesting by stainless steel mesh.		Adopt stainless steel and black double layer plastic mesh. Operates at zero energy and waste. Require dust cleaning.		2.8–3.72 L m^−2^ per day (using SFC) 2.0–3.10 L m^−2^ per day (using CFC)		– Frame and mesh material, area. – Gutter material, diameter, length, closure, position, angle of fixing. – Fog frequency, formation height, cloud base height.	^[^ ^106^ ^]^
3	The effects of surface wettability on the fog and dew moisture harvesting performance on tubular surfaces.		Comparative study of wettability under dewing and fogging conditions. Oil‐infused surfaces suitable fogging harvesting determined by water removal efficiency. Well‐wetting surfaces with lower barrier to nucleation of condensates suitable for dew harvesting determined by water capture efficiency.		In 90 min, ≈3.5–4.375 g (fog harvesting) In 90 min, ≈2.75–3.375 g (dew harvesting)		– Harvesting efficiency. – Contact angle. – Droplet mass, falling frequency. – Frequency ratio, mass ratio. – Nucleation rate, nucleation energy barrier.	^[^ ^107^ ^]^
4	Fog‐water collection for community use.		Review climatic and topographic features that determined fog formation.		Chile, Falda Verde (1.5 L m^−2^ per day) S. Africa, Soutpansberg (3 L m^−2^ per day) Ecuador, P. Grande (4 L m^−2^ per day) Guatemala, Tojquia (6 L m^−2^ per day) Canary Islands, Tenerife (10 L m^−2^ per day) Cape Verde, Serra M. (12 L m^−2^ per day)		– Amount collected. – Quality of fog water. – Impact of technology to communities.	^[^ ^98^ ^]^
5	Mist harvesting using bioinspired polydopamine coating and microfabrication technology.		Adopt biopolymer of polydopamine and negative photolithography method. Use porous membrane surfaces with contrast wettability.		97 mg cm^−2^ h^−1^		– Contact angle. – Surface free energy, surface tension, dispersive components. – Surface morphology, topography, roughness. – Water collection rate. – Wetting properties.	^[^ ^108^ ^]^
6	A facile strategy for the fabrication of a bioinspired hydrophilic‐superhydrophobic patterned surface for highly efficient fog‐harvesting.		Adopt super hydrophobically modified metal‐based gauze on the surface of hydrophilic polystyrene sheet. Can easily control the pattern's dimensions by changing the gauze mesh size and thermal pressing temperature. Low cost for replicability.		41–159 mg cm^−2^ h^−1^		– Mesh size. – Intensity, binding energy. – Water contact angle, sliding angle, droplet volume. – Copper gauge size. – Thermal treatment temperature.	^[^ ^109^ ^]^
7	A twice electrochemical‐etching method to fabricate superhydrophobic‐super hydrophilic patterns for biomimetic fog harvest.		Use electrochemical‐etching method for fabrication with boiling‐water immersion method Fabricate superhydrophobic‐superhydrophilic patterned surface. High etching potential resulted a uniform superhydrophilic dimple.		≈6000–12 000 mg h^−1^		– Etching current, voltage. – Contact angle. – Etching time. – Surface morphology, wettability. – Dimple depth. – Droplet number, mass.	^[^ ^110^ ^]^
8	Fog water collection: Challenges beyond Technology.		Review challenge and opportunity of fog collection. Standard and large fog collectors are made of polypropylene mesh nets (Raschel nets). SFC size of 1 m^2^, LFC size ranging from 40–48 m^2^. Width to height ration around 2.5–3.0.		In Tojquia, Guatemala, 28 units of LFCs provided 5000 L per day. Partial or complete failure in Serra M., Cape Verde; El Tofo & Padre H., Chile; Pachamama G., Ecuador.		– Economic factor. – Community development. – Capacity building. – Policies support. – Stakeholder involved.	^[^ ^98^, ^111^, ^112^ ^]^
9	Quantification of Fog Water Collection in Three Locations of Tenerife (Canary Islands).		– Use cylindrical wire‐harp gauges and modified Juvik‐type fog water gauges. – 46 cm high, 20 cm outer diameter. – Water funnel connected to a Rain‐O‐Matic tipping bucket gauge.		10–40 L m^−2^ per day		– Volumes, frequency of fog water. – Wind speeds, solar radiation. – Temperature inversion, relative humidity.	^[^ ^102^ ^]^
10.	Cactus kirigami for efficient fog harvesting: simplifying a 3D cactus into 2D paper art		– Adopt cactus‐inspired FC. – Spines from 3D cone to 2D triangle via wax infused kirigami. – Anisotropic shape for efficient capture of droplets. – Directional droplet self‐propulsion.		4 g cm^−2^ h^−1^ under fog flow 220 cm s^−1^		– Mass per unit area, wind flow speed, contact angle. – Spine‐to‐spine width, track‐to‐track width. – Spine density, tracks.	^[^ ^113^ ^]^

Most developing countries have installed and evaluated the potential of fog water harvesting technologies in which almost all the countries that used fog water harvesting technology are located in semi‐arid, arid regions, dry tropical as well as subtropical climates where the annual rainfall in these countries is very low. As a result, many communities from the countries constantly face a shortage of potable water outside of the rainy season and year‐round. Previous researches unveiled that countries including the driest part of the western coast of South America, the arid west coast of southern Africa, the Sub‐Sahara region of East Africa, Arabian Peninsula with dry region, the dry part of the northwest coast of Africa, semi‐arid area in Mediterranean as well as areas in southern Europe faced acute water resource problem due to hot as well as dry summer conditions, mild as well as wet winters which coupled with strong water demand for domestics, agricultural and industrial uses.^[^ ^114^ ^]^ In arid and semi‐arid regions, fog water harvesting is an alternative water source for 1 billion people which consist of one‐sixth of the human population in Earth. For example, Sahara in North Africa is the largest hot desert in the world and known as the hottest desert in the world that covered some 5.8 million km^2^. Atacama Desert in South America is the driest desert in the world which had no rain for 410 years while Namid desert in Southwest Africa is one of the most arid regions in the world with an average annual rainfall of only 18 mm.^[^ ^115^ ^]^ Through fog water harvesting technology, these areas have a water source as water is captured from fog via the condensation of water vapor during at cold nights.

Natural resources, specifically fog, that are utilized to alleviate problems of insufficient water through innovative technologies have proven to be practical and at the same time successful. Fog water harvesting technology is capable of producing an average of 15 000 L of potable tap water for 300 individuals in costal desert Chile.^[^ ^116^ ^]^ Project carried out by Schemenauer et al. had provided 800 L of water per day for two families, 31 000 L of drinking water per month and 5000 L of clean water for 27 families benefiting 127 people as well as animals.^[^ ^94^, ^112^, ^117^ ^]^ Typical water production rate of a fog collector ranges from 200 to 1000 L per day while the efficiency of the collection can be improved with larger fog droplets, higher wind speeds, and narrower collection of fiber or mesh width. Water collection rates of fog collectors are as listed in **Table** **7**.^[^ ^118^, ^119^ ^]^

**Table 7:** Water collection rates from fog collectors^[^ ^117^, ^118^ ^]^

Project	Large fog collector (LFC) water production: L/40m^2^/year (m^3^)
Gobabeb, Nambia	11 500
Chungungo, Chile	43 800
Lepelfontein, South Africa	14 700
Ilam, Nepal	52 300
Sidi Ifni, Morocco	31 800
Tenerife, Canary Islands	69 000
Meija, Peru	75 600
Hajja, Yemen	17 100
Dhofur, Oman	61 600
Chanaral, Chile	21 900
Nefasit, Eritrea	12 600
Tojquia, Guatemala	37 800

Preserving the security of water resources is the first key concern for poor rural population in South East Asia. The poor rural population in South East Asia is already facing water stresses in which many areas in the region are dependent upon limited groundwater and rainfall collection. Water is the first element that will be affected after the occurrence of climate changes through the alteration of hydrological cycle. Climate change will usually further worsen water shortage issues through extreme events such as droughts which threaten food security or extreme rainfall events which will increase the risk of flooding in different parts of the world. Water resource management will be further challenged and aggravated by sea‐level rise which eventually contributes to salt‐water intrusion into available freshwater resources.

Indonesia will most likely experience stress on water resources due to climate change. Java‐Bali regions have already faced a deficit in water balance followed by Sumatra, Sulawesi, Nusa Tenggara, and Moluccas.^[^ ^120^ ^]^ Most regions in Indonesia will suffer a gradual decrement in water supply because of the rise in temperature and changes in amount of rainfall that eventually affects the water balance. Severe water shortages might possibly occur especially in Java and Sumatra during 2020–2030 since population growth rates is estimated to increase along with increasing water demand.

Myanmar is a country rich in natural resources including water resources. Myanmar has 16% of freshwater resources in ASEAN. Almost all the rivers and streams in the country originate from the northern highlands and flow into the southern sea. Like other countries in Southeast Asia, Myanmar also suffers from impacts of climate change on water resources whereby sudden change of weather pattern directly affects the sustainability of water environment especially in the middle part of Myanmar. As agriculture is an exclusively crucial sector for economy of Myanmar, climate change will put a great risk on this sector. According to Lwin et al., rainfall pattern has changed from bimodal to unimodal distribution with a shorter duration of the rainy season from 145 to 105 days that make water scarcity become a serious issue in dry zone of Myanmar.^[^ ^121^, ^122^ ^]^

Philippines is an archipelagic nation with more than 90 million people that now face water issue due to intense climate change along with impacts on natural ecosystem such as river basins, coastal, as well as marine system and biodiversity. Independent studies on projected climate change scenarios by Philippine Atmospheric, Geophysical and Astronomical Services Administration's (PAGASA) indicated that change in climate regime will prolong dryness with prediction of reduction in rainfall in most areas in Mindanao by 2050.^[^ ^123^ ^]^

Impacts of climate change on agriculture will especially affect rice plantation sector in Thailand as Thailand was ranked as the sixth largest produces and the largest rice exporter in 2014.^[^ ^112^ ^]^ Higher environment temperature will increase evapotranspiration and thus increasing the water demand for agriculture.^[^ ^124^ ^]^ Thailand faces the worst drought in 20 years that resulted in the declining water level of the Mekong River to a lowest level in 50 years in 2010.^[^ ^125^ ^]^ Lack of rain has left as many as 17 major dams almost empty and according to the Water and Environment Institute for Sustainability, the reserved rainfall seems to be 30% lower than its average level.

## 6 Fog Collection Economic Feasibility

The collection of fog water is a simple technology in which the cost is low and the source required is sustainable for hundreds, even thousands of years. Fog water harvesting or fog collection does not any need electrical power while being relatively cheaper when compared to conventional water supply systems that require costs for fuel and spare parts as well as considered to be high maintenance system.^[^ ^114^ ^]^ Many fog water harvesting systems have been implemented in developing countries with financial support from international organizations since the developing countries face lack of technical knowledge and financial constraints on fog water harvesting system.

Fog water is collected on the mesh through the mechanisms such as impaction, direct interception, and Brownian diffusion. Fog water harvesting system utilizes two type of collectors which are standard fog collector (SFC) and large fog collector (LFC). SFC is mainly used in exploratory studies to evaluate the amount of fog water that can be collected at a specific site whereas LFC that is much larger in size has been widely used as fog collector.^[^ ^125^ ^]^ Fog collectors used worldwide are commonly made of polyethylene mesh nets or polypropylene Raschel mesh.^[^ ^116^ ^]^ The popular polyethylene and polypropylene Raschel meshes are often being utilized as material for fog water harvesting mesh because both the materials are extremely efficient in capturing fog, inexpensive, and already available as a food‐grade material while at the same time are durable enough to withstand wind, sun, UV‐radiation, as well as rain which makes them capable of rapidly draining collected water.^[^ ^114^, ^126^ ^]^ Generally, the size of SFC is 1 m × 1 m (1 m^2^) while sizes for LFC varies from 40 m^2^ (4 m × 10 m) to 48 m^2^ (4 m × 12 m) with the ratio of width to height around 2.5–3.0 m.^[^ ^126^, ^127^ ^]^ Other fabrics or materials such as nylon or aluminate greenhouse shade nets with exceptional and exclusive properties can also be utilized as a replacement for Raschel nets. Typical Raschel mesh are actually flat fibers woven into a triangular pattern with 1 mm width, 0.1 mm thickness, 10–13 mm pore size, and triangular knit that links the fibers together to prevent unravelling due to tears or wind.^[^ ^128^, ^129^ ^]^

The costs of a fog collection system are based on per m^2^ of the installed mesh and usually range from $25 to $50 per m^2^ for 2D Raschel mesh.^[^ ^98^, ^126^, ^130^ ^]^ For instance, LFC with mesh size of 40 m^2^ costs from $1000 to $2000 while 48 m^2^ LFC costs $1200 and $2400. A study by Domen et al. reported that fog water collection site can be actualized on a budget ranging from $78 to $233 USD.^[^ ^91^ ^]^ Generally, the cost for 100 LFC units is estimated to be $40 000 but depending on the access of the site and the length of pipelines where water will be delivered for the usage of the involved village.^[^ ^124^ ^]^The cost of fog to water harvesting cannot be compared with other water harvesting plants such as desalination as there is a difference in the scale of the technology. Fog harvesting technologies need very little maintenance or extra resources but can be very resource‐intense in the initial installation stage. Desalination and wastewater treatment on the other hand need constant input of energy, chemicals, and labor. The cost of LFC is dependent on the large disparity in materials and labor costs, the availability, or unavailability of subsidies, and the efficiency of the fog collector system in various locations; there is bound to be a broad range of costs that are related with the production of water through a fog collector. The price can range from $1.4 to $16.6 m^−3^.^[^ ^108^ ^]^ Calculations executed by Based on appropriate estimation on the durability of the involved equipment, meteorological conditions, and other unneglectable parameters, Cereceda et al. had proposed that fog water harvesting system costs around $1.87 m^−3^ which is one quarter of the traditional water supply around $7.25 m^−3^ where the traditional water needs to be trucked into the village. ^[^ ^131^ ^]^ Once water harvesting technology is installed, the expected life span for the polypropylene mesh in the fog water harvesting system is expected to be ≈3–10 years while 3D spacer fabric nets with greater efficiency may last for more than 20 years but the time span will vary based on the condition of environment. Although the fog collection systems are simple in terms of the design, easy to manage with minimum maintenance and operation issue, periodic and regular maintenance are vital in extending the life span of the system. The maintenance and supervision of the fog water harvesting system need to be done by the trained community. Some of the maintenance steps include the tightening loose cables, examining as well as patching any rips in the mesh, mending torn mesh while ensuring the mesh and the collection system are free of debris or algae growth.^[^ ^114^ ^]^ The annual cost for maintenance operation of fog harvesting system can range from $500 to $2500 USD depending on the local cost of labor, number of fog collectors, and repairs that need to be done.^[^ ^132^ ^]^ Poor maintenance can lead to the malfunction of the fog collectors and eventually to the end of fog collection operation. The number and size of the chosen mesh will depend on the local topography, water demand, the availability of financial resources, as well as materials along with the human capacity from the associated community to run while maintaining the fog collection system. **Table** **8** presents related projects and research in fog harvesting where its financial, economic feasibility, and model scalability are discussed.

**Table 8:** Projects and research in fog harvesting and its financial, economic feasibility and model scalability

No	Title	Financial / Economic Feasibility	Scalability	Ref
1	Exploring fog water harvesting potential and quality in the Asian region, Kingdom of Saudi Arabia.	2 m × 20 m fog collection system. 7600 SR/unit (USD 1 = SR. 3.75) Cost: 90.77 SR m^−3^ (*n* = 5, *r* = 5), 37.11 SR m^−3^ (*n* = 20, *r* = 5); *n* = life cycle, *r* = interest rate.	Life cycle of fog collector and interest rate requirement.	^[^ ^134^ ^]^
2	Fog and dew as potable water resources: maximizing harvesting potential and water quality concerns.	0.07 USD L^−1^ (bottled water: 0.22 USD L^−1^), Kutch region, Western India. 0.0011–0.0017 USD L^−1^ (trucked water: 0.0016 USD L^−1^), Chile.	Increase pH, use filters and noncorrosive piping and water storage. Disinfection requirement.	^[^ ^135^, ^136^, ^137^ ^]^
3	Fog water collection: challenges beyond technology.	Raschel mesh: 25–50 USD m^−2^ For LFC, 1000–2000 USD for 40 m^2^, 1200–2400 USD for 48m^2^ For LFC, 200 USD/unit for 40m^2^, Dar Si Hmad, Morocco (5 USD m^−2^). High efficiency 3D spacer fabric net: 830 USD m^−2^ 1.96–3.06 USD m^−3^, Atama Desert, Chile. 1.7–3.3 USD m^−3^, Eritrea.	Cost depends on the mesh material, piping, water tanks etc. Depends on its availability and price at the local area. High efficiency net can double or triple the yield, withstand wind speed of 120 km h^−1^. Mesh selection depends on durability, water draining characteristic etc. Labor and subsidies availability for LFC.	^[^ ^111^, ^132^, ^138^, ^139^, ^140^ ^]^
4	Fog‐water collection for community use.	For 60‐unit LFC with 6.2 km pipeline, 100 m^3^ storage: 37 000 USD, Chile. For 100‐unit LFC: 40 000 USD.	Cost depends on site access, piping length, product lifespan.	^[^ ^98^, ^126^, ^136^, ^141^ ^]^
5	Reviewing fog water collection worldwide and in Oman.	1.87 USD m^−3^ (water truck 7.25 USD m^−3^).	Depends on equipment durability, meteorological conditions. Cost depends on piping, distance from point of use, storing. Material depends on availability in the local market etc.	^[^ ^103^, ^132^ ^]^
6	Review of sustainable methods for atmospheric water harvesting.	400 USD for 48 m^2^ LFC. 100–200 USD for 1 m^2^ SFC. 500 USD/net for nylon net, Lima, Peru.	Depend on country and material Eiffel collector (4 × 8 × 0.3) m Harp collector (2 × 4 × 0.3) m Diagonal Harp collector (2 × 4 × 0.3) m	^[^ ^90^, ^132^, ^142^, ^143^ ^]^
7	Fog harvesting on the verge of economic competitiveness.	350 USD/module of production cost.	Require business model for enterprise and legal adaption. Eiffel collector: simplicity, high yield, reproducibility, robustness. Harp collector: best surface‐water yield ratio	^[^ ^133^ ^]^
8	Can fog and rain harvesting secure safe drinking water in rural Cameroon? – Case study of Bafou (mountainous) and Mora (low‐lying) Villages.	45 000 USD for complete Bafou's fog‐harvesting system. Including structural material, transport, labor, and maintenance.	Prepare multiple alternative water sources during drought. Assessment of microbiology and chemistry experts is desirable on water quality.	^[^ ^144^ ^]^
9	Fog as a Fresh‐water resource: overview and perspectives	150 USD for SFC.	Depend on local market situation. Cost covers piping, shipping, storage, transport. Might require meteorology and water supply expert.	^[^ ^104^ ^]^
10	Cactus kirigami for efficient fog harvesting: simplifying a 3D cactus into 2D paper art	0.5 USD m^−2^ for construction cost.	Use paper‐based substrate. Simplified structure, tunable and scalable. Low‐cost materials, bioinspired interfaces.	^[^ ^113^ ^]^

Commonly, the type of mesh that is often used in most fog collectors is Raschel shade net material. The Raschel shade net mesh is made from food safe polyethylene material and of a fiber width that seems to be effective in collecting fog droplets. New mesh designs have been developed depending on the fog collection sites such as location with the presence of strong wind that will cause fog collectors system to fail.^[^ ^145^ ^]^ Currently, Raschel mesh (35% shading) based fog collection nets have been successfully applied for many years in 35 countries in five continents. Mesh of different materials have been tested for sites with windy and extreme conditions.^[^ ^146^ ^]^ Fog harvesting system with double layered robust material, stainless mesh, and co‐knitted with poly material has been employed in South Africa. Fog water collection mesh with 3D net structure is currently still under development in Germany. 3D meshes will bring about increasing yields due to greater surface areas as well as the effectiveness of the meshes at draining water and at the same time are more efficient than the standard 2D Raschel mesh when employed at location whereby the winds are blowing parallel to the fog collector.

**Figure 5:**
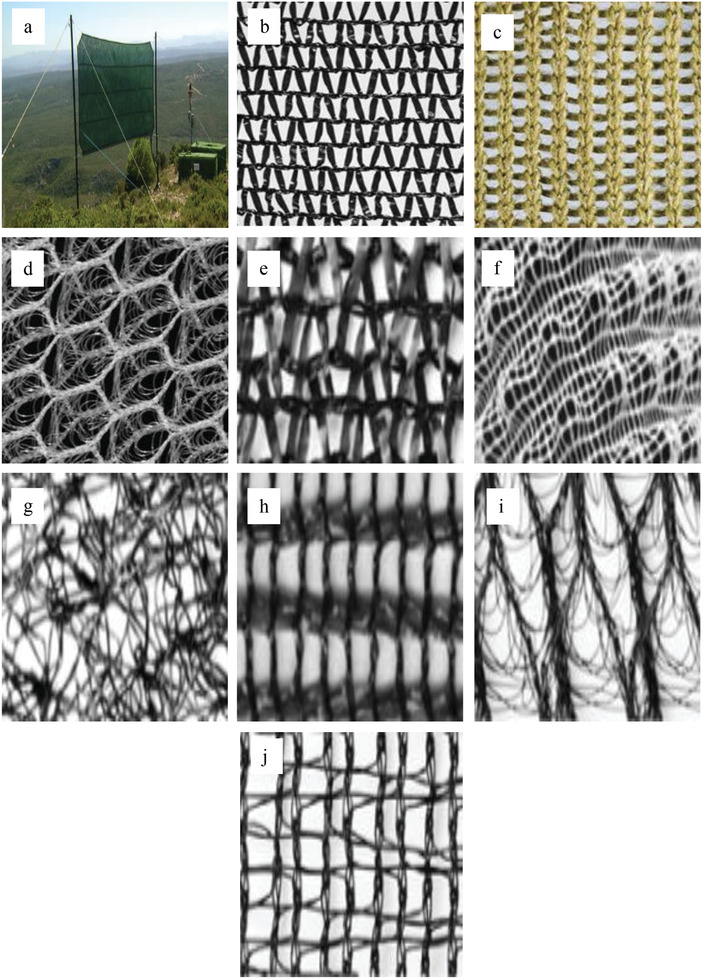
a) 18 m^2^ flat‐panel fog collector at Mount Machos with the water tanks; b) single layer Rashel mesh (35% shading); c) stainless steel poly‐yarn mesh used in South Africa; d) 3D prototype poly‐material mesh; e) Raschel net (PP), double‐layered; f) Dimple dot fabric (PES); g) Enkamat (PA6); h) Shade net (HDPE); i) Spacer fabric (PES); j) Hail protection net (HDPE), double layered. Reproduced with permission.^[^ ^104^ ^]^ Copyright 2014, Springer Nature. Reproduced with permission.^[^ ^118^ ^]^ Copyright 2015, Martina Mayerhofer.

In contrast, the cost as well as the availability of 3D meshes may possibly prohibit and prevent the usage of 3D meshes.^[^ ^91^ ^]^ Munich industrial designer, Peter Trautwein designed the new “CloudFisher” that is capable of generating a large amount of drinking water and is more stable in strong winds than the nets used previously around the world.^[^ ^121^ ^]^ Six different types of nets (Raschel net, Dimple dot fabric, Enkamat, Shade net, Spacer fabric, and Hail protection net) were tested at the same time for the stability and yield of the nets at Moroccan highlands. The test phase was concluded in July 2015. All the utilized materials are characterized via UV‐resistance since the force of the wind is not the only parameter that should be considered but the solar radiation also cannot to be underestimated. **Figure** **5** shows the mesh and fog water harvesting system which has been used in the previous research.

The most important factors determining the feasibility of implementing fog water collection system are the expected yield and the quality of the harvested water.^[^ ^94^ ^]^ Quality of the water collected through fog collectors needs to be checked before being supplied for human consumption or for any usage. The quality of harvested fog water relies on the composition of the incoming fog, the material of collectors, as well as the chemical compositions of dry deposition formed on the surface of the fog collectors.^[^ ^147^ ^]^ There are several aspects of water chemistry that are important in fog collection system such as the chemical composition of the fog in the air before the fog strikes any collecting surface, the chemistry of the water collected in an LFC and the potential alteration in chemical composition of harvested fog water while being stored in storage tanks.^[^ ^116^ ^]^ In addition, fog water quality will also be affected by urban and industrial emissions. Generally, fog water has a higher concentration of soluble particles than rainwater mainly due to the droplet‐forming process that often occurs at a lower elevations whereby the quality of the air is greatly affected by human activities.^[^ ^116^ ^]^ Typical contaminations in fog water include low levels of total dissolved solids such as calcium, sodium, chloride, and bicarbonate. In general, results collected from different countries reveal that harvested fog water samples meet the respective standards for water quality of national and World Health Organization.^[^ ^103^, ^116^, ^135^, ^147^, ^148^ ^]^ The studies indicated that the quality of water harvested through fog water collection system met the WHO standards for ion and 23 heavy metals with a slightly lower pH value and can be used for drinking water and domestic and agricultural purposes. Generally, harvested water from fog collector is safe for drinking and seems suitable for all purpose.

## 7 Conclusion

Climate change could dramatically affect human life and lead to violent conflict. Less developed countries are generally more affected than the industrialized countries. The impacts of climate change on Southeast Asia include prolonged water demand and water scarcity. Fog water harvesting can provide great alternative and is considered as potential source for water in order to supply communities having only little annual rainfall and shortages of water while at the same time help to reduce dependence on traditional water sources such as groundwater in Southeast Asia. Over the last decades, water harvesting technology benefited human race as the technique managed to help to supply water to developing and rural regions while operating in arid and semi‐arid regions to harvest water for usage in domestic, agricultural, and reforestation programs. Under minimal maintenance and low operational issues, fog water harvesting is an environmental friendly intervention that does not rely on energy consumption. Fog water harvesting system should be considered as a master plan for water supply for countries in Southeast Asia.

Fog harvesting might not be the most suitable solution for water scarcity in urban area. However, the feasibility statement of this paper exhibits a significant impact to the vulnerable and disadvantaged community specially the regions with vulnerable index ranging from 0.68 to 1.00. Fog‐to‐water technology bridges the water gap especially in drought frequent areas with annual drought frequency of around 0.455 and above. The greatest threat of climate change in these areas is the change in intensity and patterns of rainfall that caused prolonged drought season. In this context, the conventional rain harvesting, solar powered water pumping and water filtration might not be the most meaningful solution to low income community. During prolonged drought season where there is no rain, fog‐to‐water is the close‐at‐hand fresh water source. It also makes significant difference to sparse population in many arid regions specially the archipelago nations in SEA. During severe drought, the untapped water resources from dry air can be useful for drinking, crop irrigation, livestock beverage, and forest restoration especially the dryland mountain and coastal areas. In long term, such water deficit gap can be narrowed, improving the water security, food security, crop productivity, sanitation etc. Hence, promoting public health, welfare, and life expectancy for those experienced a much higher level of complexity of water scarcity issues.

## Conflict of Interest

The authors declare no conflict of interest.
